# Evaluation of the Microbial Load and Heavy Metal Content of Two Polyherbal Antimalarial Products on the Ghanaian Market

**DOI:** 10.1155/2020/1014273

**Published:** 2020-05-15

**Authors:** Bernard K. Turkson, Merlin L. K. Mensah, George H. Sam, Abraham Y. Mensah, Isaac K. Amponsah, Edmund Ekuadzi, Gustav Komlaga, Emmanuel Achaab

**Affiliations:** ^1^Institute of Traditional and Alternative Medicine, University of Health and Allied Sciences, Ho, Ghana; ^2^Department of Herbal Medicine, Faculty of Pharmacy and Pharmaceutical Sciences, Kwame Nkrumah University of Science and Technology, Kumasi, Ghana; ^3^Department of Pharmacognosy, Faculty of Pharmacy and Pharmaceutical Sciences, Kwame Nkrumah University of Science and Technology, Kumasi, Ghana; ^4^Tafo Government Hospital, Ghana Health Service, Accra, Ghana

## Abstract

The use of herbal products has increased and become more popularized globally; however, limited studies coupled with questions related to the quality and safety of these herbal products have been raised. Herbal products with hope of their nontoxicity may play a role of alternative to overcome the problems of multi-drug resistant pathogens. Medicinal plants used as raw materials for production may have quality and safety issues due to proximity to wastewater application of fungicides and pesticides, which may be directly deposited superficially or absorbed by the plant system. Therefore, possible contamination of some Ghanaian herbal products cannot be ignored, as it may severely affect human life in the process of treatment. *Aim*. To evaluate the microbial load and the presence of toxic heavy metals in *Mist Amen Fevermix* and *Edhec Malacure*, two polyherbal products used in the treatment of uncomplicated malaria in Ghana. *Methods*. Thermo Elemental M5 Atomic Absorption Spectrophotometer (AAS) fitted with graphite furnace and an auto sampler was used to determine the heavy metal contents of the herbal products. The herbal samples were evaluated for the microbial load by using the appropriate culture media. *Results and Analysis*. *Mist Amen Fevermix and Edhec Malacure* complied with the safety limits evaluated for all different microbial counts and contamination. The following heavy metals were present in *Mist Amen Fevermix and Edhec Malacure Mixture*: Fe, Ni, K, Zn, Hg, Cu, Mn, Cr, Cd, Pb, Fe, Cu, K, and Na. Ni was below detectable limit in *Edhec Malacure*. *Conclusion*. *Mist Amen Fevermix and Edhec Malacure* may be assured of safety. The products contained heavy metals, but all were within acceptable limit established by the FAO/WHO. The levels of microbial contamination were below the maximum acceptable limit.

## 1. Introduction

The use of herbal products for treating various diseases, including malaria, predates the history of mankind. Herbal therapies are mainstream for nearly all illnesses [1]. Herbal products use has increased globally; they are in high demand in both developed and developing countries for primary health care purposes. This is a result of the attributed wide range of biological activities, perceived safety, ready availability, and lesser cost associated with the products. Due to increased occurrence of falsified medicines, incidence of adverse drug reactions, drug resistance by various diseases and the economic burden of orthodox medicines, public, academic, and government interests in herbal products as alternatives has grown exponentially due to their acceptability and effectiveness [2].

Most distributors and consumers consider herbal products to be safe, although toxic heavy metals and microbial contamination in finished herbal products have been a concern. Medicinal herbal products have been reported to be contaminated with toxic heavy metals and microorganisms found in the soil and plants where they were grown [3]. Heavy metals and microbial contamination of herbal products pose a threat to their quality and safety. Normally, the shortcomings of herbal products include unhygienic conditions under which they are produced [4, 5]. The contaminants that present serious health hazards are pathogenic bacteria such as *Salmonella spp*, *Escherichia coli*, *Staphylococcus aureus,* and *Shigella spp* [6]. Inappropriate cleaning, unsuitable transportation, and poor storage conditions render the medicinal plant materials vulnerable to infestations and expose them to much microbial contamination during the production stage, leading to deterioration in quality. This may give rise to the risk of mycotoxin production, especially aflatoxin, which has been proven to be mutagenic, carcinogenic, teratogenic, neurotoxic, nephrotoxic, and immunosuppressive [7, 8].

Quality and safety parameters of herbal medicines based on the heavy metal contents and microbial load have been an important concern for health authorities and health professionals. The contamination of these herbal products reduces their effectiveness and also poses serious health hazards to consumers. Heavy metals, if consumed, can accumulate in different organs of the body, leading to unwanted side effects [6, 9]. Therefore, it is important to evaluate the heavy metal content and microbial load of herbal products based on relevant scientific guidelines [10].

In Ghana, several herbal products are readily available on the market, and about 75% of the population relies on herbal medicines for their primary health care needs. However, about 9% of the population depends on herbal products for the treatment of malaria [11]. However, studies on them regarding, especially, heavy metal contents are limited. Therefore, it is important to evaluate for the toxic heavy metal and microbial content with the aim to establish the level of microbes and heavy metals present in *Mist Amen Fevermix* and *Edhec Malacure*, two finished herbal products formulated and used in Ghana for the management of uncomplicated malaria.

## 2. Materials and Methods

### 2.1. Reagents, Glassware, and Instrumentation

Two bottles each of *Mist Amen Fevermix* and *Edhec Malacure*, Thermo Elemental M5 Atomic Absorption Spectrophotometer (AAS), (Model ICE3000; Thermo Scientific, USA) fitted with graphite furnace and an auto sampler at the Faculty of Agriculture, Department of Soil Science laboratory, Kwame Nkrumah University of Science and Technology (KNUST), concentrated HNO_3_, concentrated HCl, H_2_O, nutrient agar, MacConkey agar, Sabouraud agar, *Salmonella* agar, *Shigella* Agar and potato dextrose agar laboratory incubator (Gallenkamp), oven (Gallenkamp), electrical balance (Mettler Toledo), and general laboratory glass wares were obtained from the central stores of the Department of Microbiology, KNUST, Ghana.

### 2.2. Samples

Two bottles each of *Mist Amen Fevermix* and *Edhec Malacure* were obtained from the herbal medicine unit of the Tafo Government Hospital, Kumasi, Ghana. *Mist Amen Fevermix* is a finished herbal product registered with the FDA, Ghana. *Mist Amen Fevermix* is produced by Amen Scientific Herbal Hospital and is on the recommended essential herbal medicines list of the Ministry of Health and used in the herbal medicine units in Ghana. *Edhec Malacure Mixture* is a finished herbal product, registered by the FDA, Ghana. *Edhec Malacure* is produced by Edu Herbal Clinic, Mankessim, Central Region, Ghana. It is not on the recommended essential herbal medicines list; however, it is sold on the market.

### 2.3. Heavy Metals Determination

Test for elemental constituents of the herbal products were determined using the Thermo Elemental M5 Atomic Absorption Spectrophotometer (AAS) (Model ICE3000; Thermo Scientific, USA), fitted with graphite furnace and an auto sampler at the Faculty of Agriculture, Department of Soil Science laboratory, Kwame Nkrumah University of Science and Technology (KNUST). Eleven heavy metals were analyzed in each product. An aliquot of 1 mL from two samples each of *Mist Amen Fevermix* and *Edhec Malacure* were placed in a 250 mL beaker, and 5 mL each of freshly prepared acid mixture of concentrated HNO_3_, concentrated HCl, and H_2_O in the ratio 1.5 : 0.5 : 0.5 was added. The mixture was gently heated on a hot plate maintaining a temperature of 150°C until the sample had completely dissolved to give a clear solution. During the digestion process, the inner walls of the beaker were washed with deionized water to prevent sample loss. After digestion, the *Mist Amen Fevermix* and *Edhec Malacure* were made up to 50 mL with deionized water and analyzed. Multielement standard solutions of all the elements involved were prepared by diluting 1000 mg/L stock solutions with 5 percent nitric acid solution [10]. Samples were analyzed in duplicate and the average was calculated.

### 2.4. Microbial Load Determination

Analysis for the microbial load of *Mist Amen Fevermix* and *Edhec Malacure* were determined using nutrient agar, MacConkey agar, Sabouraud agar, *Salmonella* agar, *Shigella* Agar and potato dextrose agar which were bought from Lab Chem Medical Supplies, Kumasi. The microbial load of *Mist Amen Fevermix* and *Edhec Malacure* were assessed in the microbiology laboratory of the Department of Microbiology, Kwame Nkrumah University of Science and Technology (KNUST). Samples were analyzed in duplicate and the average was used.

### 2.5. Preparation of Culture Media

*Mist Amen Fevermix* and *Edhec Malacure* were shaken vigorously to ensure uniform distribution of microorganisms if any. An aliquot of 5 mL each of the two antimalarial products was pipetted into 95 ml of sterile distilled water for stock sample preparation and was subjected to tenfold serial dilution in sterile test tubes. An aliquot of 1 mL each of stock sample was aseptically transferred and mixed in 9 ml of sterile distilled water. All media used were prepared according to the manufacturer's specifications. For total viable bacterial and coliform count, the appropriate solutions were transferred into sterile duplicate plates, and 20 mls each of nutrient and MacConkey agar were added and mixed separately. For fungal count, 1 mL each of *Mist Amen Fevermix* and *Edhec Malacure* were streaked over duplicate plates of prepared dried potato dextrose agar. Plates were incubated at 32°C for 48 hours for total bacterial counts, at 37°C for 24 hours for coliform counts, and at 25°C for 5 days for fungi counts. Plates were counted for total bacterial and fungal counts [12].

## 3. Results

The results for heavy metals tested in *Mist Amen Fevermix* and *Edhec Malacure* are stated in Table 1. The results showed that all the heavy metals in *Mist Amen Fevermix* and *Edhec Malacure* were within the permissible limits and some were not yet established [13, 14].

The results of microbial load evaluated in *Mist Amen Fevermix* and *Edhec Malacure* are recorded in Tables 2 and 3 below. The results showed that the microbial contamination in *Mist Amen Fevermix* and *Edhec Malacure* were below the maximum acceptable limit.

## 4. Discussion

There has been an increased use of herbal products globally including Ghana. An herbal medicine services have been integrated into the health-care delivery system since the year 2011 and appears to have led to increased demand for herbal medicines. Despite the upsurge in usage, herbal products have not been subjected to rigorous quality assurance to benefit patrons and satisfy critics of these herbal remedies.

Also, national limits for toxic heavy metals and the microbial contamination in various types of herbal products are different for each country and depend on the herb type and whether it is raw material or finished herbal product [10]. Even though there are no permissible limits for toxic heavy metals and the microbial load for herbal products established by the Ghana Standards Authority, Ghana, the Food and Drugs Authority, has the mandate to withdraw any herbal products proven to be unsafe for consumption based on reports of suspected adverse reaction or reactions, or problems related to hazards or harms [15]. The increased use of herbal products in Ghana warrants that the products used are free of toxic heavy metals and the microbial contamination in tests and they are of good quality and safe.

The elemental analysis showed that *Mist Amen Fevermix* and *Edhec Malacure Mixture* contain heavy metals such as presented in Table 1. The heavy metals were all present and within permissible limits set by FAO/WHO for herbal products [16, 17]. However, nickel was below detectable limits in the *Edhec Malacure Mixture*. The concentrations of iron, copper, potassium, and sodium were more in *Edhec Malacure Mixture* than in *Mist Amen Fevermix*, but no regulatory limits have been established for these metals in herbal medicines by the WHO [14]. The results and observations are in line with previous studies which found that heavy metals found in Ghanaian, Egyptian, Indian, and Pakistani herbal preparations and medicinal plants are relatively low [18, 19].

The levels of the elements analyzed in *Mist Amen Fevermix* were within FAO/WHO limits [20]; a finding that is very important for the quality control of the products because of the long-term safety implications for users who may get exposed to excessive amounts of these elements [13]. Despite the fact that the concentrations of the metals present in the test products were low and within the permissible limit, dosages above the critical limit may lead to possible heavy metal toxicity.

Contamination of herbal products by microorganism can be attributed to many causes including: environmental pollution and soil contamination [21]. The microbiological results, in Tables 2 and 3, shows that *Salmonella*, *Shigella*, *Pseudomonas*, and *E*. *coli* were not detected in the study products; however, a total aerobic viable count of 1.27 × 10^3^ cfu/mL was detected in *Mist Amen Fevermix*, and a count of 2.17 × 10^3^ cfu/mL was detected in *Edhec Malacure*. These microbial counts are below the maximum permissible limit of 1.0 × 10^5^ cfu/mL. Also, the amount of yeast and molds in *Mist Amen Fevermix* was 1.09 × 10^3^ cfu/mL and *Edhec Malacure Mixture* had 1.83 × 10^3^ cfu/mL. The microbes present in *Mist Amen Fevermix* and *Edhec Malacure Mixture* were below the acceptable maximum limit of 1.0 × 10^7^ cfu/mL. This observation is in line with previous studies which found that pathogenic bacteria like *Salmonella spp* and *Shigella* were not isolated from some herbal preparations [22], as in the case of *Mist Amen Fevermix* and *Edhec Malacure Mixture* due to good manufacturing practices observed. However, some herbal antidiabetic preparations formulated in Bangladesh were contaminated with microorganisms which pose a potential risk for human health, and care should be taken in every step involved in the preparation of herbal preparations to assure safety [23].

## 5. Conclusion

Evaluation of the heavy metal contents and microbial load of *Mist Amen Fevermix* and *Edhec Malacure* revealed that *Mist Amen Fevermix* and *Edhec Malacure* contain toxic heavy metals, but within the acceptable limits. Also, the microbes in *Mist Amen Fevermix* and *Edhec Malacure* herbal products were within acceptable limits. This is an indication that the products may be safe for use in the treatment of uncomplicated malaria infection. This data could provide a reference to the field of herbal preparations in Ghana.

## Figures and Tables

**Table 1:** Elemental analysis of *Mist Amen Fevermix* and *Edhec Malacure Mixture*.

Metals (mg/kg)	Samples								Permissible limits (mg/kg)
	Mist Amen Fevermix				Edhec Malacure				
	A	B	AVE	SD	A	B	AVE	SD	
As	0.062	0.086	0.074	0.012	0.007	0.003	0.005	0.002	5.0 [13]
Cu	0.010	0.016	0.013	0.003	5.235	3.532	4.3835	0.8515	Not set [14].
Cd	0.009	0.005	0.007	0.002	0.08	0.02	0.05	0.03	0.3 [13]
Fe	0.09	0.066	0.078	0.012	23.559	26.721	25.14	1.581	Not set [14].
Hg	0.013	0.009	0.011	0.002	0.00084	0.00122	0.00103	0.00019	0.5 [13]
Mn	0.22	0.35	0.285	0.065	2.22184	2.3965	2.30917	0.08733	Not set [14]
Ni	0.008	0.002	0.005	0.003	BDL	BDL	BDL	BDL	1.683 [13]
Pb	0.001	0.017	0.009	0.008	0.00025	0.00269	0.00147	0.00122	10 [13]
Zn	0.102	0.076	0.089	0.013	0.422	0.438	0.430	0.008	27.4 [13]
K	3.97	3.69	3.83	0.14	305.172	406.322	355.747	50.575	Not set [14]
Na	0.37	0.88	0.625	0.255	41.215	38.9956	40.1053	1.1097	Not set [14]

A and B samples of *Mist Amen Fevermix* and *Edhec Malacure*; AVE:average; SD:standard deviation.

**Table 2:** Microbial load of *Mist Amen Fevermix*.

Test	Results				Acceptable limits
	A	B	AVE	SD	
Total aerobic viable count (NA; 37°C; 24 hrs) ≤1 × 10^5^ cfu/mL	1.21 × 10^3^	1.33 × 10^3^	1.27 × 10^3^	0.06	Not more 1.0 × 10^7^ cfu/mL
Test for S*almonella*, *Shigella*. BSA/37°C/48 hrs. Nil/L	0	0	0	0	Absent
Test for *Escherichia coli*. MAC/37°C/48 hrs. Nil/L	0	0	0	0	Not more 1.0 × 10^2^ cfu/mL
Test for *Pseudomonas cetrimide* PCA/37°C/48 hrs. Nil/L	0	0	0	0	Absent
Test for yeasts and molds. PDC/SAB/25°C/5 days	1.01 × 10^3^	1.17 × 10^3^	1.09 × 10^3^	0.08	Not more 1.0 × 10^5^ cfu/mL

A and B samples of *Mist Amen Fevermix*; AVE: average; SD: standard deviation.

**Table 3:** Microbial load of *Edhec Malacure*.

Test	Results				Acceptable limits
	A	B	AVE	SD	
Total aerobic viable count (NA; 37°C; 24 hrs) ≤1 × 10^5^ cfu/mL	2.11 × 10^3^	2.23 × 10^3^	2.17 × 10^3^	0.06	Not more 1.0 × 10^7^ cfu/mL
Test for *Salmonella*, *Shigella*. BSA/37°C/48 hrs. Nil/L	0	0	0	0	Absent
Test for *Escherichia coli*. MAC/37°C/48 hrs. Nil/L	0	0	0	0	Not more 1.0 × 10^2^ cfu/mL
Test for *Pseudomonas cetrimide* PCA/37°C/48 hrs. Nil/L	0	0	0	0	Absent
Test for yeast and molds. PDC/SAB/25°C/5 days	1.23 × 10^3^	2.43 × 10^3^	1.83 × 10^3^	0.6	Not more 1.0 × 10^5^ cfu/mL

A and B samples of *Edhec Malacure*; AVE: average; SD: standard deviation.

## Data Availability

The data used to support the findings of this study are available from the corresponding author upon request.

## References

[1] Barakat E. M. F., El Wakeel L. M., Hagag R. S. (2013). Effects of Nigella sativaon outcome of hepatitis C in Egypt. *World Journal of Gastroenterology*.

[2] Parmar S., Shah N., Kasarwala M., Virpura M., Shah K., Patel P. (2011). Determination of quercetin by HPTLC method present in zymodyne syrup—a poly herbal formulation. *International Journal of Pharmaceutical Sciences and Research*.

[3] Keter L., Too R., Mwikwabe N. (2016). Bacteria contaminants and their antibiotic sensitivity from selected herbal medicinal products from eldoret and mombasa, Kenya. *American Journal of Microbiology*.

[4] Oyetayo V. O. (2008). Microbial load and antimicrobial property of two Nigerian herbal remedies. *African Journal of Traditional, Complementary and Alternative Medicines*.

[5] Nwoko O. C., Mgbeahuruike L. (2011). Heavy metal contamination of ready-to-use herbal remedies in south eastern Nigeria. *Pakistan Journal of Nutrition*.

[6] Ngari W. F., Gikonyo K. N., Wanjau R. N., Njagi E. N. M. (2013). Investigation of selected pathogenic microorganisms and toxic elements in herbal materials used in management of oral health in nairobi county, Kenya. *Journal of Applied Environ Mental and Biological Sciences*.

[7] Ashiq S., Hussain M., Ahmad B. (2014). Natural occurrence of mycotoxins in medicinal plants: a review. *Fungal Genetics and Biology*.

[8] Wu H.-C., Wang Q., Yang H.-I. (2009). Aflatoxin B1 exposure, hepatitis B virus infection, and hepatocellular carcinoma in taiwan. *Cancer Epidemiology Biomarkers & Prevention*.

[9] Macdonald I., Uvo Oghale O., Jimoh A. (2015). Heavy metals contamination of some poly herbal products from lagos state, Nigeria. *Journal of Ayurvedic and Herbal Medicine*.

[10] World Health Organization (2007). *Guidelines for Assessing Quality of Herbal Medicines with Reference to Contaminant and Residue*.

[11] Bugyei K., Boye G., Addy M. (2010). Clinical efficacy of a tea-bag formulation of cryptolepis sanguinolenta root in the treatment of acute uncomplicated falciparum malaria. *Ghana Medical Journal*.

[12] Feng P., Weagant S., Grant M., Burkhardt W. (2002). Bacteriological analytical manual.in Chapter 4. *Enumeration of Escherichia coli and the Coliform Bacteria*.

[13] FAO/WHO (1984). Contaminants. *Codex Alimentarius*.

[14] Ulla R., Khader J. A., Hussain I., AbdElsalam N. M., Talha M., Khan N. (2012). Investigation of macro and micro-nutrients in selected medicinal plants. *African Journal of Pharmacy and Pharmacology*.

[15] FDA, 2012, http://www.moh.gov.gh/foods-and-drug-authority/

[16] World Health Organization (2005). *Quality Control Methods for Medicinal Plant Materials*.

[17] World Health Organization (2006). *Guideline: Potassium Intake for Adults and Children*.

[18] Abou-Arab A. A. K., Abou Donia M. A. (2000). Heavy metals in Egyptian spices and medicinal plants and the effect of processing on their levels. *Journal of Agricultural and Food Chemistry*.

[19] Jabeen S., Shah M. T., Khan S., Hayat M. Q. (2010). Determination of major and trace elements in ten important folk therapeutic plants of Haripur basin, Pakistan. *Journal of Medicinal Plants Research*.

[20] Turkson Bernard K., Kofi P. O., Achaab E. (2015). Clinical evaluation of the safety and effectiveness of mist amen Fevermix, a Ghanaian Bi-herbal product, used in the management of uncomplicated malaria. *Journal of Natural Sciences Research*.

[21] Ahmad I. Z., Ahmad A., Mabood A., Tabassum H., Khan M. I. R., Khan N. A. (2017). Effects of different metal stresses on the antioxidant defense systems of medicinal plants. *Reactive Oxygen Species and Antioxidant Systems in Plants: Role and Regulation under Abiotic Stress*.

[22] Walther C., Marwa K. J., Jeremiah S. (2016). Microbial contamination of traditional liquid herbal medicinal products marketed in Mwanza city: magnitude and risk factors. *Pan African Medical Journal*.

[23] Zamir R., Hosen A., Obayed Ullah M., Nahar N. (2015). Microbial and heavy metal contaminant of antidiabetic herbal preparations formulated in Bangladesh. *Evidence-Based Complementary and Alternative Medicine*.

